# Supplementary material for: Population-Based Analysis of National Comprehensive Cancer Network (NCCN) Guideline Adherence for Patients with Anal Squamous Cell Carcinoma in California
Source: Cancers (Basel). 2023 Feb 25;15(5):1465. doi: 10.3390/cancers15051465 (PMC10000877; doi:10.3390/cancers15051465)
Supplement: Supplementary file 1 [file cancers-15-01465-s001.zip › cancers-2178346-supplementary.pdf]

Supplementary Materials

Table S1. Patient characteristics by treatment adherent status. .

	**Total**	**Non-adherent care**	**Adherent care**	
	**N (Column %)**	**N (Row %)**	**N (Row %)**	*p* **value** [a]
Total	4740 (100.0)	926 (19.5)	3814 (80.5)	
Age at diagnosis				<0.0001
18–44	303 (6.4)	61 (20.1)	242 (79.9)	
45–54	1100 (23.2)	172 (15.6)	928 (84.4)	
55–64	1158 (32.9)	252 (16.2)	1306 (83.8)	
65+	1779 (37.5)	441 (24.8)	1338 (75.2)	
Year of diagnosis				0.1876
2004–2009	1592 (33.6)	328 (20.6)	1264 (79.4)	
2010-2017	3148 (66.4)	598 (19.0)	2550 (81.0)	
Gender				<0.0001
Male	1576 (33.2)	366 (23.2)	1210 (76.8)	
Female	3159 (66.6)	560 (17.7)	2599 (82.3)	
Race/ethnicity				0.1467
Non-Hispanic white	3632 (76.6)	688 (18.9)	2944 (81.1)	
Non-Hispanic black	301 (6.4)	70 (23.3)	231 (76.7)	
Hispanic	603 (12.7)	125 (20.7)	478 (79.3)	
Asian	125 (2.6)	22 (17.6)	103 (82.4)	
Others/Unknown	79 (1.7)	21 (26.6)	58 (73.4)	
Insurance				<0.0001
Managed care	2234 (47.1)	375 (16.8)	1859 (83.2)	
Medicare	1196 (25.2)	298 (24.9)	898 (75.1)	
Medicaid	429 (9.1)	89 (20.7)	340 (79.3)	
Other Insurance (FFS, Tricare, VA or NOS)	674 (14.2)	111 (16.5)	563 (83.5)	
Not insured or unknown	207 (4.4)	53 (25.6)	154 (74.4)	
Socioeconomic Status (SES)				<0.0001
Lowest SES	667 (14.1)	173 (25.9)	494 (74.1)	
Lower-middle SES	887 (18.7)	186 (21.0)	701 (79.0)	
Middle SES	991 (20.9)	183 (18.5)	808 (81.5)	
Higher-middle SES	1154 (24.3)	198 (17.2)	956 (82.8)	
Highest SES	1041 (22.0)	186 (17.9)	855 (82.1)	
Marital status				<0.0001
Single, separated, divorced, widowed, unmarried or unknown	2869 (60.5)	628 (21.9)	2241 (78.1)	
Married	1871 (39.5)	298 (15.9)	1573 (84.1)	
Tumor stage				<0.0001
I	953 (20.1)	279 (29.3)	674 (70.7)	
II	1682 (35.5)	330 (19.6)	1352 (80.4)	
III	1686 (35.6)	229 (13.6)	1457 (86.4)	
IV	419 (8.8)	88 (21.0)	331 (79.0)	
Grade or differentiation of the tumor				<0.0001
Grade I or well differentiated	438 (9.2)	108 (24.7)	330 (75.3)	
Grade II or moderately well differentiated	1474 (31.1)	317 (21.5)	1157 (78.5)	
Grade III or poorly differentiated	1396 (29.5)	210 (15.0)	1186 (85.0)	
Grade IV or undifferentiated/anaplastic	50 (1.1)	14 (28.0)	36 (72.0)	
Grade and differentiation not stated	1382 (29.2)	277 (20.0)	1105 (80.0)	

[a] Chi square test for the difference between non-adherent group and adherent group.

Table S2. Adjusted odds ratios from logistic regression on receiving NCCN guideline adherent care.

	**Odds ratio and 95% C.I.**			***p*-value**
Age at diagnosis (years)	0.969	0.962	0.976	<0.0001
Year of diagnosis	1.01	0.99	1.03	0.2591
Female	1.38	1.18	1.62	<0.0001
Race/ethnicity				
Non-Hispanic white		Reference Group		
Non-Hispanic black	0.77	0.57	1.03	0.08
Hispanic	0.88	0.70	1.11	0.2899
Asian	1.13	0.70	1.84	0.6116
Others/Unknown	0.59	0.35	1.00	0.0484
Insurance				
Managed care		Reference Group		
Medicare	0.86	0.71	1.03	0.1077
Medicaid	0.75	0.57	1.00	0.0476
Other Insurance (FFS, Tricare, VA or NOS)	0.88	0.69	1.13	0.3206
Not insured or unknown	0.60	0.43	0.85	0.0041
Socioeconomic Status (SES)				
Lowest SES	0.65	0.50	0.83	0.0007
Lower-middle SES	0.82	0.64	1.04	0.0935
Middle SES	0.98	0.77	1.23	0.8343
Higher-middle SES	1.06	0.84	1.33	0.6284
Highest SES		Reference Group		
Marital status				
Single, separated, divorced, widowed, unmarried or unknown		Reference Group		
Married	1.35	1.14	1.58	0.0004
Tumor stage				
I		Reference Group		
II	1.82	1.50	2.20	<0.0001
III	2.64	2.15	3.24	<0.0001
IV	1.65	1.24	2.20	0.0006
Grade or differentiation of the tumor				
Grade I or well differentiated		Reference Group		
Grade II or moderately well differentiated	0.99	0.76	1.29	0.9479
Grade III or poorly differentiated	1.52	1.15	2.00	0.0032
Grade IV or undifferentiated/anaplastic	0.76	0.39	1.51	0.4355
Grade and differentiation not stated	1.11	0.85	1.44	0.4514

Table S3.: Survival Analysis of DSS using Cox regression model and hazard ratios for covariates in multivariate model with and without adherence.

	Disease specific survival							
	**Adjusted hazard ratio and 95% C.I.**			***p*-value**	**Adjusted hazard ratio and 95% C.I.**			***p*-value**
Age at diagnosis (years)	1.022	1.013	1.031	<0.0001	1.019	1.010	1.028	<0.0001
Year of diagnosis	0.99	0.96	1.02	0.5067	0.99	0.97	1.02	0.6914
Female	0.71	0.58	0.87	0.0008	0.73	0.59	0.89	0.0021
Race/ethnicity								
Non-Hispanic white	Reference Group				Reference Group			
Non-Hispanic black	1.40	0.98	1.99	0.0623	1.37	0.97	1.95	0.0784
Hispanic	0.90	0.66	1.23	0.5048	0.90	0.66	1.23	0.5118
Asian	0.91	0.51	1.62	0.7358	0.92	0.52	1.64	0.7805
Others/Unknown	0.80	0.36	1.81	0.5971	0.79	0.35	1.77	0.5655
Insurance								
Managed care	Reference Group				Reference Group			
Medicare	0.92	0.72	1.18	0.5024	0.90	0.71	1.16	0.4234
Medicaid	1.11	0.80	1.54	0.542	1.11	0.80	1.54	0.5237
Other Insurance (FFS, Tricare, VA or NOS)	0.94	0.69	1.27	0.6761	0.94	0.69	1.28	0.6895
Not insured or unknown	1.01	0.66	1.56	0.954	0.96	0.62	1.48	0.8482
Socioeconomic Status (SES)								
Lowest SES	1.14	0.81	1.62	0.4511	1.08	0.77	1.53	0.6532
Lower-middle SES	1.45	1.07	1.94	0.0151	1.43	1.06	1.93	0.0186
Middle SES	1.20	0.89	1.61	0.2355	1.19	0.88	1.60	0.2537
Higher-middle SES	1.09	0.82	1.46	0.5534	1.08	0.81	1.45	0.5964
Highest SES	Reference Group				Reference Group			
Marital status								
Single	Reference Group				Reference Group			
Married	0.85	0.69	1.04	0.1131	0.87	0.71	1.07	0.1857
Tumor stage								
I	Reference Group				Reference Group			
II	2.20	1.47	3.29	0.0001	2.41	1.61	3.61	<0.0001
III	4.28	2.90	6.32	<0.0001	4.96	3.35	7.35	<0.0001
IV	15.16	10.04	22.90	<0.0001	17.53	11.55	26.58	<0.0001
Grade or differentiation of the tumor								
Grade I or well differentiated	Reference Group				Reference Group			
Grade II or moderately well differentiated	1.60	1.04	2.47	0.0316	1.64	1.07	2.52	0.0244
Grade III or poorly differentiated	1.66	1.08	2.56	0.0208	1.75	1.14	2.69	0.0112
Grade IV or undifferentiated/anaplastic	2.43	0.99	5.96	0.0529	2.24	0.91	5.51	0.078
Grade and differentiation not stated	1.12	0.72	1.74	0.6283	1.15	0.74	1.79	0.5402
Received NCCN adherent care								
No	-	-	-	-	1.96	1.56	2.46	<0.0001
Yes					Reference Group			

Table S4. Survival Analysis of OS using Cox regression model and hazard ratios for covariates in multivariate model with and without adherence.

	Overall survival							
	**Adjusted hazard ratio and 95% C.I.**			***p*-value**	**Adjusted hazard ratio and 95% C.I.**			***p*-value**
**Age at diagnosis (years)**	1.038	1.033	1.043	<0.0001	1.035	1.030	1.040	<0.0001
Year of diagnosis	1.00	0.99	1.02	0.7396	1.01	0.99	1.03	0.3653
Female	0.60	0.54	0.67	<0.0001	0.62	0.56	0.69	<0.0001
Race/ethnicity								
Non-Hispanic white	Reference Group				Reference Group			
Non-Hispanic black	1.61	1.33	1.94	<0.0001	1.61	1.33	1.94	<0.0001
Hispanic	0.97	0.82	1.14	0.6924	0.96	0.81	1.14	0.6579
Asian	0.97	0.71	1.32	0.8205	0.98	0.72	1.33	0.8732
Others/Unknown	0.86	0.54	1.37	0.5126	0.83	0.52	1.33	0.4458
Insurance								
Managed care	Reference Group				Reference Group			
Medicare	1.34	1.18	1.52	<0.0001	1.33	1.17	1.51	<0.0001
Medicaid	1.52	1.27	1.83	<0.0001	1.53	1.27	1.83	<0.0001
Other Insurance (FFS, Tricare, VA or NOS)	0.93	0.77	1.12	0.4405	0.93	0.77	1.12	0.4276
Not insured or unknown	1.15	0.90	1.47	0.2662	1.11	0.87	1.42	0.393
Socioeconomic Status (SES)								
Lowest SES	1.66	1.38	1.99	<0.0001	1.56	1.30	1.88	<0.0001
Lower-middle SES	1.49	1.26	1.78	<0.0001	1.48	1.25	1.76	<0.0001
Middle SES	1.28	1.08	1.52	0.0049	1.28	1.08	1.51	0.0052
Higher-middle SES	1.24	1.05	1.46	0.013	1.24	1.05	1.47	0.0113
Highest SES	Reference Group				Reference Group			
Marital status								
Single	Reference Group				Reference Group			
Married	0.84	0.75	0.94	0.0027	0.86	0.77	0.97	0.0121
Tumor stage								
I	Reference Group				Reference Group			
II	1.84	1.53	2.21	<0.0001	2.00	1.67	2.41	<0.0001
III	2.59	2.16	3.12	<0.0001	2.99	2.48	3.60	<0.0001
IV	8.60	7.01	10.54	<0.0001	9.76	7.95	11.98	<0.0001
Grade or differentiation of the tumor								
Grade I or well differentiated	Reference Group				Reference Group			
Grade II or moderately well differentiated	1.24	1.01	1.53	0.039	1.28	1.04	1.57	0.0216
Grade III or poorly differentiated	1.24	1.00	1.53	0.0462	1.31	1.06	1.61	0.0126
Grade IV or undifferentiated/anaplastic	1.94	1.19	3.16	0.0078	1.84	1.13	3.00	0.0144
Grade and differentiation not stated	1.09	0.88	1.35	0.4272	1.13	0.92	1.40	0.2541
Received NCCN adherent care								
No	-	-	-	-	1.87	1.66	2.12	<0.0001
Yes					Reference Group			

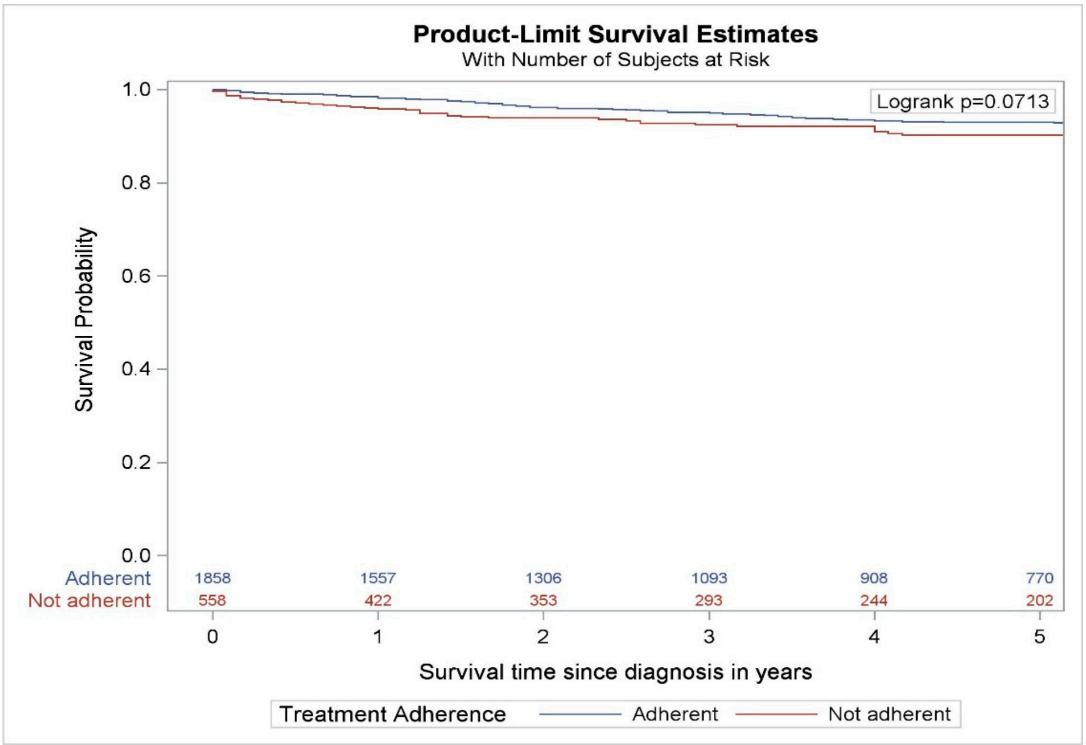

A

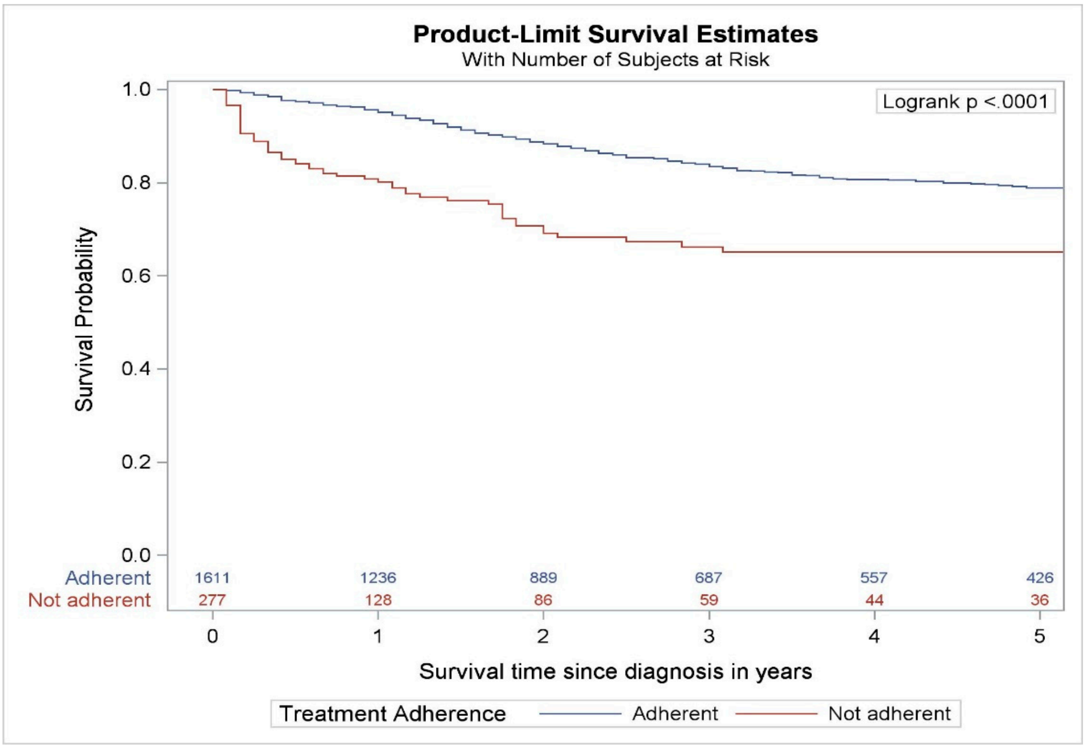

B

Figure S1. Disease-specific survival graph with log rank test for (**A**) early-stage disease (Stage I or Stage II), (B) for late-stage disease (Stage III or Stage IV).

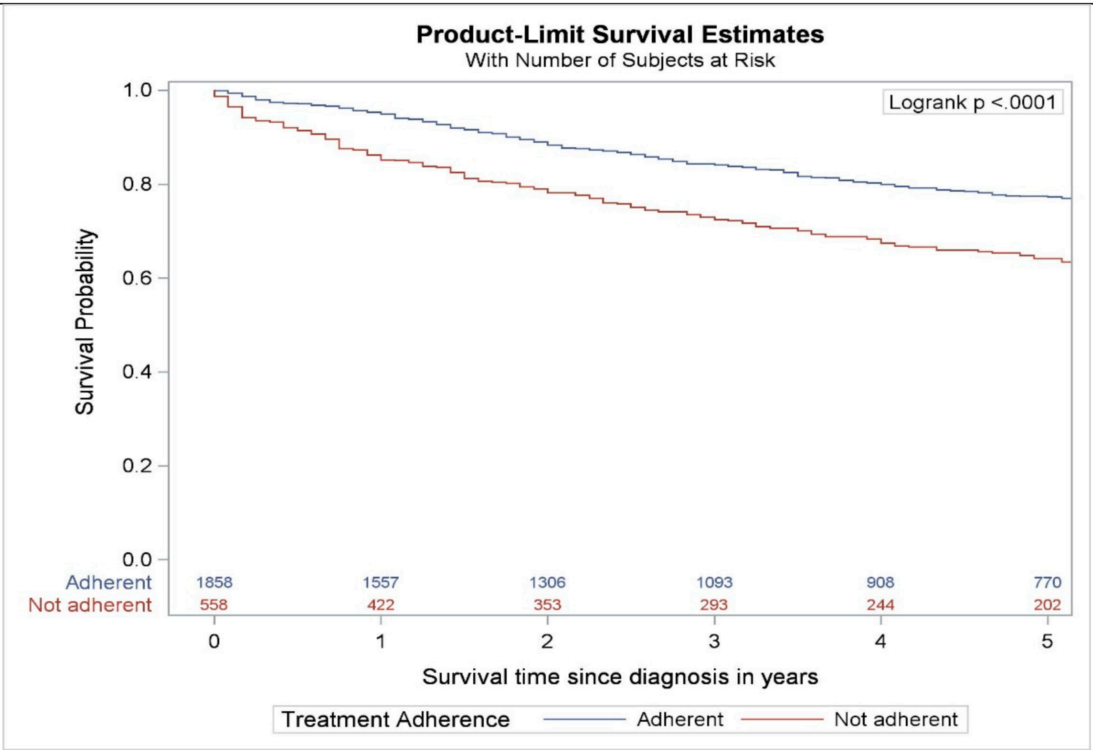

A

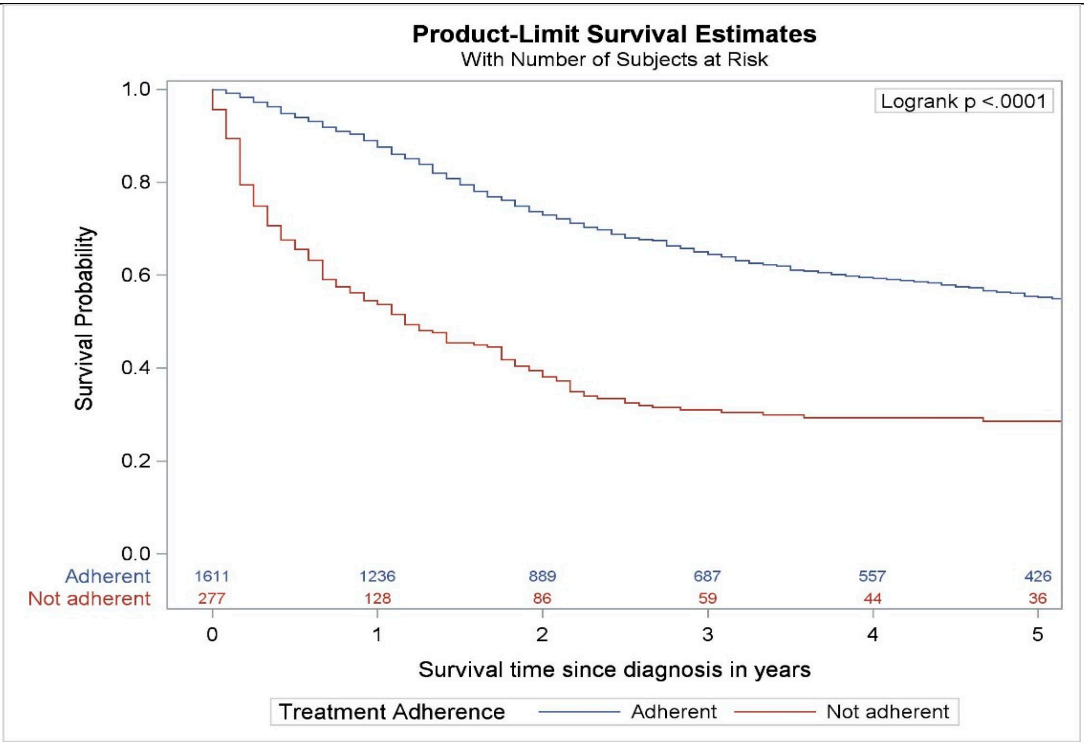

B

Figure S2. Overall survival graph with log rank test for (A) early-stage disease (Stage I or Stage II), (B) for late-stage disease (Stage III or Stage IV).
